# Advanced Deep Learning Fusion Model for Early Multi-Classification of Lung and Colon Cancer Using Histopathological Images

**DOI:** 10.3390/diagnostics14202274

**Published:** 2024-10-12

**Authors:** A. A. Abd El-Aziz, Mahmood A. Mahmood, Sameh Abd El-Ghany

**Affiliations:** Department of Information Systems, College of Computer and Information Sciences, Jouf University, Sakaka 72388, Saudi Arabia; mamahmood@ju.edu.sa (M.A.M.); saabdelwahab@ju.edu.sa (S.A.E.-G.)

**Keywords:** colon, ResNet-101V2, NASNetMobile, EfficientNet-B0, deep learning, HIs, features fusion, fine-tuning, tumor, cancer

## Abstract

Background: In recent years, the healthcare field has experienced significant advancements. New diagnostic techniques, treatments, and insights into the causes of various diseases have emerged. Despite these progressions, cancer remains a major concern. It is a widespread illness affecting individuals of all ages and leads to one out of every six deaths. Lung and colon cancer alone account for nearly two million fatalities. Though it is rare for lung and colon cancers to co-occur, the spread of cancer cells between these two areas—known as metastasis—is notably high. Early detection of cancer greatly increases survival rates. Currently, histopathological image (HI) diagnosis and appropriate treatment are key methods for reducing cancer mortality and enhancing survival rates. Digital image processing (DIP) and deep learning (DL) algorithms can be employed to analyze the HIs of five different types of lung and colon tissues. Methods: Therefore, this paper proposes a refined DL model that integrates feature fusion for the multi-classification of lung and colon cancers. The proposed model incorporates three DL architectures: ResNet-101V2, NASNetMobile, and EfficientNet-B0. Each model has limitations concerning variations in the shape and texture of input images. To address this, the proposed model utilizes a concatenate layer to merge the pre-trained individual feature vectors from ResNet-101V2, NASNetMobile, and EfficientNet-B0 into a single feature vector, which is then fine-tuned. As a result, the proposed DL model achieves high success in multi-classification by leveraging the strengths of all three models to enhance overall accuracy. This model aims to assist pathologists in the early detection of lung and colon cancer with reduced effort, time, and cost. The proposed DL model was evaluated using the LC25000 dataset, which contains colon and lung HIs. The dataset was pre-processed using resizing and normalization techniques. Results: The model was tested and compared with recent DL models, achieving impressive results: 99.8% for precision, 99.8% for recall, 99.8% for F1-score, 99.96% for specificity, and 99.94% for accuracy. Conclusions: Thus, the proposed DL model demonstrates exceptional performance across all classification categories.

## 1. Introduction

According to the World Health Organization (WHO), cancer is one of the most lethal and widespread diseases globally, second only to heart disease in terms of its impact on public health. Cancer refers to conditions in which the body produces abnormal cells due to random mutations. Cancer cells grow independently, become genetically unstable, are characterized by the uncontrolled growth and division of abnormal cells, and spread throughout the organs. The American Cancer Society projects that over 606,000 individuals will pass on from the cancer disease. In addition, more than 1.8 million new cases were accounted for in 2020. The brain, breasts, lungs, colon, liver, rectum, skin, prostate, and stomach are the most frequently cancer-affected organs [1].

Cancer can be caused by various factors, including genetic, biological, and physical carcinogens like ultraviolet rays and radiation exposure, smoking, and alcohol consumption, as well as behavioral characteristics like a high body mass index (BMI). However, the reason may differ from patient to patient. Fatigue, pain, difficulty breathing, persistent cough, nausea, weight loss, bleeding, bruising, and muscle pain are all common signs of cancer. Firstly, patients frequently have few or no symptoms, and most of the time, symptoms do not show up until it is too late. None of the symptoms are exclusive to cancer patients [1,2].

Lung cancer causes 18.4% of cancer-related deaths and 11.6% of all cancer cases, whereas colon cancer accounts for 9.2% of all deaths caused by cancer worldwide. The simultaneous occurrence of both lung and colon cancers, known as synchronous lung and colon cancers, is a relatively uncommon occurrence, at about 17% [3]. Although this is rare, metastasis, the spread of cancer cells between the lung and colon, is extremely common without an early diagnosis [4]. Also, colon cancer accounts for 9.2% of all cancer-related deaths worldwide [5,6]. Malignant tumor rates have been increasing worldwide in ongoing patterns, which could be credited to an expansion in the population. All people can get cancer; however, people aged 50 to 60 are more likely to get it. If current trends continue, passing rates could increase by 60% in 2035, as certain evaluations indicate [7,8].

Lung cancer, like many other types of cancer, often begins when normal lung cells undergo genetic mutations that cause them to grow and divide uncontrollably. These mutated cells can then form tumor clusters or masses within the lungs [9]. The most significant factors contributing to the global rise in cancer incidence are the increased number of older people and increased lung exposure to hazardous substances. It is difficult to treat these conditions because their symptoms typically do not show up until they have spread to other organs [10].

Non-smokers can also be affected by lung cancer, but the risk is higher for smokers. There are two main types of lung cancer: Non-small-cell lung cancer (NSCLC), which includes adenocarcinoma and squamous cell carcinoma, and small-cell lung cancer (SCLC) [11].

NSCLC can occur in people who currently smoke, have a smoking history, or have recently quit smoking. While smoking is a major risk factor for lung cancer, including adenocarcinoma, this type of lung cancer is often associated with a broader range of risk factors. It is less related to smoking. Adenocarcinoma of the lung typically originates in the cells that line the smaller airways of the lungs and can form tumors in the lung tissue. It is primarily affecting young women.

SCLC is commonly associated with a history of smoking. It often develops in individuals who are current smokers or have a history of tobacco use, and it is strongly linked to cigarette smoking as a major risk factor. It can develop in any part of the lungs. It grows and spreads so quickly that it is difficult to treat [12,13].

Tumors in the rectum or colon often originate when healthy cells in the lining of the gastrointestinal tract’s inner wall (the mucosa) begin to undergo uncontrolled growth and division. This uncontrolled growth can lead to the formation of a mass or lump, which is commonly referred to as a tumor. Most of the time, this kind is malignant [14]. Colon adenocarcinomas are the most common type of colorectal cancer, and they usually originate in the epithelial cells that line the inner surface of the large intestine (colon and rectum). Mucinous adenocarcinoma and signet ring cell adenocarcinoma are two less common subtypes of adenocarcinoma. However, their extreme aggressiveness makes them hard to treat [15]. Your age, ethnicity, financial situation, smoking habits, gender, and other factors can all affect your body’s age. However, mutations can occur as quickly as several months if an individual has a rare genetic condition [16].

One way to reduce cancer mortality is by early diagnosis and providing the appropriate patient treatment. When cancer is diagnosed early, the patient’s chances of recovery and survival rates are increased. Diagnostic techniques such as MRI, PET scan, CT scan, ultrasound, and biopsy are vital in detecting cancer cells, assessing the extent of cancer, and aiding in treatment choices. To diagnose and identify the various types and subtypes of cancer, experienced pathologists must examine the microscopic histopathology slides during the biopsy [3]. HIs are crucial in determining a patient’s likelihood of survival and are widely used by medical professionals for diagnosis. Traditionally, health professionals undergo a lengthy procedure to diagnose cancer by examining HIs. However, the technological tools that are currently available make it possible to carry out this procedure in less effort and time [3].

The traditional diagnostic methods for lung and colon cancer detection, despite their widespread use, come with several limitations in terms of accuracy, invasiveness, cost, and early detection. These limitations include [17]:

1. **Chest X-rays and CT scans for Lung Cancer:**
  - **Low Sensitivity for Early Detection**: Chest X-rays and CT (computed tomography) scans, commonly used for lung cancer screening, often fail to detect small or early-stage tumors, limiting their ability to identify cancer at an early, more treatable stage.
  - **Radiation Exposure**: Both X-rays and CT scans expose patients to radiation, which can accumulate with repeated screenings and potentially increase cancer risk, especially in patients requiring long-term monitoring.
  - **False Positives**: These imaging techniques can sometimes show benign lesions or abnormalities (like scar tissue or infections) that mimic cancer, leading to unnecessary biopsies, anxiety, or treatments.
  - **Lack of Specificity:** CT scans can detect abnormal lung masses but cannot definitively determine whether they are cancerous. Further invasive procedures, such as biopsies, are required for confirmation.
2. **Sputum Cytology for Lung Cancer:**
  - **Limited Effectiveness for Small or Peripheral Tumors**: Sputum cytology (the examination of mucus for cancer cells) is often ineffective at detecting tumors that are located in the outer parts of the lungs.
  - **Low Sensitivity**: The test has a high chance of missing cancer cells, particularly in the early stages. It is also less effective in detecting non-small-cell lung cancers.
3. **Biopsy for Lung Cancer:**
  - **Invasiveness**: A biopsy, where a tissue sample is removed from the lung for testing, is an invasive procedure that can result in complications like bleeding or infection.
  - **Delay in Diagnosis**: Biopsy results can take time to process, leading to delays in diagnosis and treatment initiation.
4. **Colonoscopy for Colon Cancer:**
  - **Invasiveness and Discomfort**: Colonoscopy is the most common method for diagnosing colon cancer, but it is an invasive procedure that requires bowel preparation and sedation, and involves risks such as bowel perforation, bleeding, and infection.
  - **Patient Reluctance**: Due to the invasive nature and preparation requirements, many patients avoid or delay undergoing a colonoscopy, which can prevent early detection.
  - **False Negatives**: In some cases, small polyps or flat lesions may be missed during a colonoscopy, especially if the bowel preparation is inadequate.
5. **Sigmoidoscopy Colon Cancer:**
  - **Limited Coverage**: Sigmoidoscopy only examines the lower part of the colon, potentially missing cancers in the upper regions of the colon (proximal cancers).
  - **Invasiveness**: Although less invasive than a full colonoscopy, it still involves discomfort and carries some risk of complications like bleeding or perforation.

The shortcomings of conventional diagnostic methods underscore the necessity for more sophisticated, non-invasive, and sensitive diagnostic technologies. Emerging methods, such as molecular imaging, liquid biopsies, and artificial intelligence (AI)-powered diagnostic tools, are being developed to address these limitations by enabling earlier and more accurate detection of lung and colon cancers.

AI technologies have demonstrated tremendous potential in recent years and provided us with a viable alternative to conventional methods. They have gained a reputation for the speed at which they analyze data and make decisions. Machine learning (ML) is used in many ways in pathology, like finding diseases and creating intelligent systems that prescribe conventional medicines based on a patient’s symptoms [18]. Several types of biomedical data have been classified and predicted using ML methods. Machines can analyze high-dimensional data like images using DL techniques. DL, a subset of ML, deals with algorithms based on the brain’s anatomy and function. Recently, colon and lung cancer diagnosis utilizing DL techniques has become an increasingly popular area of research [19].

This paper proposes a robust fine-tuned DL model of ResNet-101V2, NASNetMobile, and EfficientNet-B0 for multi-classification of colon and lung cancer. The ImageNet dataset was used to pre-train ResNet-101V2, NASNetMobile, and EfficientNet-B0. We used the GlobalAveragePooling2D simultaneously to smooth all layers into a feature vector by obtaining the average for each input channel. Moreover, we used a concatenate layer to fuse all of the pre-trained individual feature vectors of ResNet-101V2, NASNetMobile, and EfficientNet-B0 into a single feature vector. We fine-tuned the fusion single feature vector on the training set of the LC25000 dataset.

The proposed fine-tuned DL model aims to assist pathologists in efficiently de-tecting colon and lung cancer. This model has significant potential to be a valuable tool in pathology, thereby providing appropriate treatment for patients. The proposed DL model has the potential to reduce the effort, time, and cost for pathologists while enhancing early detection and treatment. 

The proposed DL model was evaluated on the LC25000 dataset, which contains HIs of the lungs and colon. Normalization and resizing techniques were used for pre-processing the LC25000 dataset. The proposed model achieved 99.8%, 99.8%, 99.8%, 99.9%, and 99.99% for precision, recall, F1-score, specificity, and accuracy, respectively. Therefore, the proposed DL model delivered cutting-edge performance across all classes. A summary of the research’s contributions is provided below:

We propose a fine-tuned DL model based on ResNet-101V2, NASNetMobile, and EfficientNet-B0 to detect lung and colon cancer accurately. 

We used the feature fusion process to help with learning the features of HIs for the depiction of their rich inner data.

The proposed fine-tuned DL model was not overfitted. 

We compared the proposed fine-tuned model to more recent convolutional neural network (CNN) models for a highly accurate multi-classification. 

The proposed fine-tuned model could detect colon and lung cancers with high accuracy and less effort and time. It will help pathologists to detect colon and lung cancers early, hence providing appropriate treatment for patients.

The proposed fine-tuned model achieved 99.8%, 99.8%, 99.8%, 99.9%, and 99.99%, for precision, recall, F1-score, specificity, and accuracy.

The remaining sections of this research are structured as follows: Section 2 provides an overview of the existing research on diagnosing lung and colon diseases. Section 3 offers a comprehensive explanation of the model’s materials and architecture. The implementation and evaluation of the proposed DL model are discussed in Section 4. Section 5 provides the conclusion of the research.

## 2. Literature Review

A. H. Chehade1 et al. [1] used five ML methods: random forest (RF), support vector machine (SVM), eXtreme gradient boosting (XGBoost), linear discriminant analysis (LDA), multilayer perceptron (MLP), feature engineering, and image processing techniques to classify the HIs of lung and colon cancer of the LC25000 dataset. They used an image enhancement technique, unsharp masking, to pre-process the dataset. The obtained experimental results demonstrated that ML models accurately identify classes of lung and colon cancer subtypes and produce satisfactory results. With an F1-score and an accuracy of 98.8% and 99%, respectively, the XGBoost model performed the best.

M. Ali and R. Ali [20] proposed an improved computerized-aided diagnosis (CAD) system for predicting lung and colon adenocarcinomas and squamous cell carcinomas. They developed the CAD system using a capsule network with multiple inputs and digital HIs. They used standard convolutional layers (CLs) in the convolutional layer block (CLB), whereas they used separable CLs in the separable CLB. The CAD system was measured on the LC25000 dataset. The authors pre-processed the dataset using gamma, multi-scale fusion, color balancing, correction, and image sharpening. The accuracy and F1-score of the proposed CAD were 99.58% and 99.04%, respectively; hence, it delivered cutting-edge performance across all classes for colon and lung irregularities.

Sakr, A.S. et al. [21] proposed a new lightweight DL method based on a CNN. The authors pre-processed the LC25000 dataset by a normalization technique. The analysis of the results showed that the proposed DL for detecting colon cancer had an F1-score, precision, recall, and accuracy of 99.49%, 99%, 100%, and 99.50%, respectively.

M. Masud et al. [22] analyzed the HIs of five distinct types of colon and lung tissues, two benign and three malignant, and proposed a CNN classification framework. They used the LC25000 dataset for training and validation of the proposed method. They used two domain transformations to extract four sets of features for image muti-classification. The results demonstrated that the accuracy and F1-score of the proposed CNN classification framework were 96.33% and 96.38%, respectively, for identifying cancerous tissues.

Naresh Kumar et al. [23] compared two methods for feature extraction of the multi-classification tasks of the LC25000 dataset. The first method executed six handcrafted feature extraction methods based on color, texture, shape, and structure. The authors trained and tested four ML techniques: SVM-RBF, RF, MLP, and gradient boosting (GB) for the multi-classification of lung and colon cancers. In the second method, the authors used transfer learning for training seven DL methods for deep feature extraction from HIs with lung and colon cancers. The deeply extracted features were input attributes in SVM-RBF, MLP, RF, and GB techniques to classify lung and colon cancers. According to the findings, the proposed method achieved excellent precision, accuracy, ROC-AUC, F1-score, and recall. The RF model can identify the colon and lung cancer tissue using deep features extracted from DenseNet-121 with a recall and accuracy of 98.60%, F1-score of 98.5%, ROC-AUC of 01, and precision of 98.63%.

M. Shahid et al. [24] used AlexNet and tuned it over the LC25000 colon and lung cancer HI dataset. The proposed model was efficient and accurate for rapid colon and lung cancer diagnosis. Before training ALexNet on the LC25000 dataset, its four layers were altered. The HIs of the LC2500 dataset were pre-processed using resizing, normalizing, and data augmentation methods. With an overall accuracy of 98.4%, the implemented methodology has proven computationally efficient.

Md. A. Talukder et al. [25] proposed a hybrid ensemble model based on a feature extraction approach to detect colon and lung cancers. The proposed model used ensemble techniques and feature extraction. VGG16, VGG1, MobileNet, DenseNet169, and DenseNet201 were used for feature extraction. The authors used the LC25000 dataset to evaluate the model. The accuracy of the hybrid model was 99.30%, 100%, and 99.05% for detecting lung and colon, colon, and lung cancer, respectively.

O. Attallah et al. [26] proposed a framework for detecting lung and colon cancers. The proposed framework used MobileNet, SqueezeNet, and ShuffleNet models to extract features of the images from the LC25000 dataset. The extracted features from the DL models were reduced by principal component analysis (PCA), a feature selection technique, and the fast Walsh–Hadamard transform. Hence, the discrete wavelet transform combined reduced features from the fast Walsh–Hadamard. Moreover, the PCA features are combined. Finally, the reduced features from PCA, the fast Walsh–Hadamard, and the discrete wavelet were fed to several ML techniques. The accuracy of the proposed framework was 99.6%.

J. D. Akinyemi et al. [27] proposed a computer-aided diagnostic (CAD) system based on the EfficientNet model to detect lung and colon cancers. The LC25000 dataset was utilized to evaluate the EfficientNet model. The accuracy of EfficientNet was 99.72%.

In our proposed model, we combined the integrative features from ResNet-101V2, NASNetMobile, and EfficientNet-B0 to enhance the detection of colon and lung tumors. Each model has its strengths in extracting different types of features due to their unique designs. This fusion reduces the weaknesses of individual models while maximizing their strengths, resulting in improved generalization. ResNet-101V2: This model is known for its deep structure and use of residual connections, making it effective at capturing high-level semantic features. NASNetMobile: This model is designed for mobile devices, offering a balance between accuracy and computational efficiency. EfficientNet-B0: This model employs a compound scaling method, allowing it to achieve top-tier performance with fewer parameters.

By merging ResNet-101V2, NASNetMobile, and EfficientNet-B0:

- We created a more comprehensive set of features that can capture both global and local image details across multiple scales and resolutions. This improves the model’s ability to detect both small, subtle tumors (early-stage cancer) and large, well-defined ones (advanced-stage cancer).
- The combination of these networks reduces the likelihood of missing important features or making incorrect classifications, thus boosting both sensitivity and specificity in detecting lung and colon tumors.
- By combining multi-scale features, the integrated model can detect tumors of various sizes and appearances, which is crucial in medical imaging, where tumors can be highly variable.
- This hybrid approach ensures that the final model can be deployed in real-world clinical settings, including resource-constrained environments, without sacrificing performance.
- Combining features from these pre-trained models allows for faster convergence and better performance when adapted to lung and colon cancer detection.
- The integrative model can learn from existing knowledge while adapting to the unique characteristics of medical images.
- The integrated model is better suited to handle class imbalance, leading to improved performance in detecting rare cancerous lesions in large datasets dominated by healthy samples.

## 3. Materials and Methods

### 3.1. Materials

**Figure 1:**
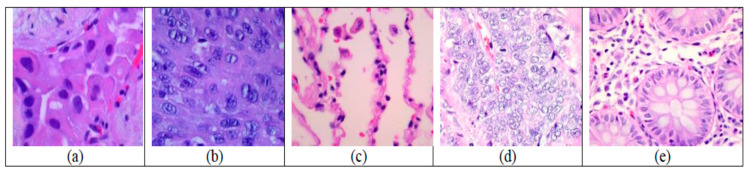
40× Tissue samples of the LC25000 dataset: (**a**) NSCLC, (**b**) SCLC, (**c**) benign lung tissue, (**d**) colon cancer tissue, and (**e**) benign colon tissue.

This research used the LC25000 HI dataset to train and evaluate the proposed DL model. The LC25000 HIs dataset divided colon and lung cancer tissues into five categories: benign lung tissues (lung_b_t), adenocarcinoma (NSCLC) (lung_aca), squamous cell carcinoma (SCLC) (lung_scc), benign colonic tissues (colon_b_t), and colon adenocarcinoma (colon_aca). The HIs of the LC25000 dataset were taken by Andrew A. Borkowski et al. [28]. It has 25,000 HIs, of which 15,000 are for the three lung classes and 10,000 are for the two colon classes. Each class of the five classes has 5000 images [23]. Figure 1 depicts samples from the LC25000 HI dataset.

Pathology slides contained 1250 images of cancer tissues, with 250 images for each class. Andrew A. Borkowski et al. [28] implemented data augmentation by left and right rotations (with a probability of 1.0 up to 25 degrees) and vertical and horizontal flips (with a probability of 0.5) to bring the dataset up to 25,000 HIs. The original 1024 × 768 pixels were resized to 768 × 768 pixels to reshape the images to a square shape [23].

Colon adenocarcinoma represents over 95% of colon cancers. It is caused by a polyp (tissue growth) known as an adenoma, which later develops into cancer and is mostly found in the large intestine. The most prevalent lung cancer type is lung adenocarcinoma (NSCLC), which accounts for over 40% of all lung cancer cases. It first develops primarily in epithelial cells before spreading to the lungs’ alveoli. The second type of lung cancer is squamous cell carcinoma (SCLC), which accounts for nearly 30% of all cases. The bronchi, or airways of the lungs, are where this cancer grows. Although benign colon and lung tumors are typically not life-threatening, surgical removal must be recommended [23].

Table 1 presents the features of the five classes: benign lung tissues, NSCLC, SCLC, benign colonic tissues, and colon cancer tissue [29]:

Cancerous tissues, such as NSCLC, SCLC, and colon cancer, exhibit several distinct features compared to their benign counterparts. Nuclear atypia: Cancerous tissues display a high degree of abnormality in the appearance and structure of the cell nuclei, which are the control centers of the cells. Mitotic activity: Cancerous tissues show an increased and disorganized pattern of cell division, with an abnormal number of cell divisions occurring. Structural disorganization: Cancerous tissues have a disrupted and disorganized internal structure, in contrast to the normal, uniform cell structure observed in benign lung and colon tissues. These differences in nuclear appearance, cell division, and overall tissue organization are key characteristics that distinguish cancerous tissues from their benign counterparts.

### 3.2. Methodology

**Figure 2:**
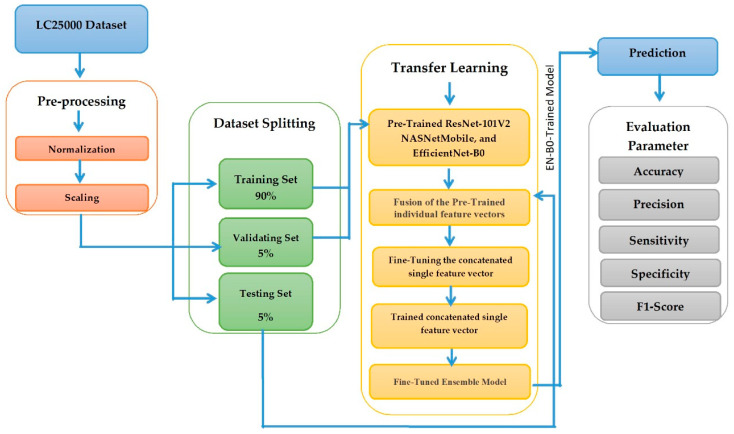
The steps of the proposed DL model.

We proposed a DL model using three CNN models: ResNet-101V2, NASNetMobile, and EfficientNet-B0. We chose the three DL models for their high performance in analyzing high-dimensional data like images. Each CNN model of ResNet-101V2, NASNetMobile, and EfficientNet-B0 needed to be revised regarding the varieties of texture and shape of the input HI images. However, the proposed model of the three DL models resulted in a notable accomplishment in multi-classification, as it effectively utilized the strengths of each model to enhance the overall accuracy. The proposed model’s objective is to analyze the HIs of the LC25000 dataset to detect colon and lung cancers. The phases of the proposed model of the three DL models are depicted in Figure 2. The proposed DL model consists of the following phases:

**Phase 1: The LC25000 dataset pre-processing**: The LC25000 dataset [28] was downloaded from the Kaggle environment and pre-processed in phase 1. Pre-processing is essential in dealing with the results’ accuracy because it effectively yields accurate results. HIs were pre-processed by normalization and resizing methods.

**Phase 2: The LC25000 dataset division**: In phase 2, the LC25000 dataset was split into 90% (11250 HIs) as a training set, 5% (625 HIs) as a test set, and 5% (625 HIs) as a validation set.

**Phase 3: Pre-training for the ResNet-101V2, NASNetMobile, and EfficientNet-B0 models**: The three CNN models were pre-trained on the ImageNet dataset. In phase 3, we executed the supervised pre-training phase of the transfer learning process. We trained ResNet-101V2, NASNetMobile, and EfficientNet-B0 on the ImageNet dataset. The pre-trained models used GlobalAveragePooling2D simultaneously to smooth all layers into a feature vector by obtaining the average for each input channel.

**Phase 4: Fusion of the pre-trained individual feature vectors**: In phase 4, a concatenate layer was used to concatenate all of the pre-trained individual feature vectors of ResNet-101V2, NASNetMobile, and EfficientNet-B0 into a single feature vector.

**Phase 5: Fine-tuning the concatenated single feature vector**: In phase 5, we fine-tuned the concatenated single feature vector and trained it on the training set of the LC25000 dataset. With this concatenation, more features can be caught; hence, the accuracy of the fine-tuned technique was increased.

**Phase 6: The proposed model evaluation**: Finally, the proposed technique was measured on the test set of the LC25000 dataset using the measured metrics, like accuracy, F1-score, sensitivity, and precision.

**Figure 3:**
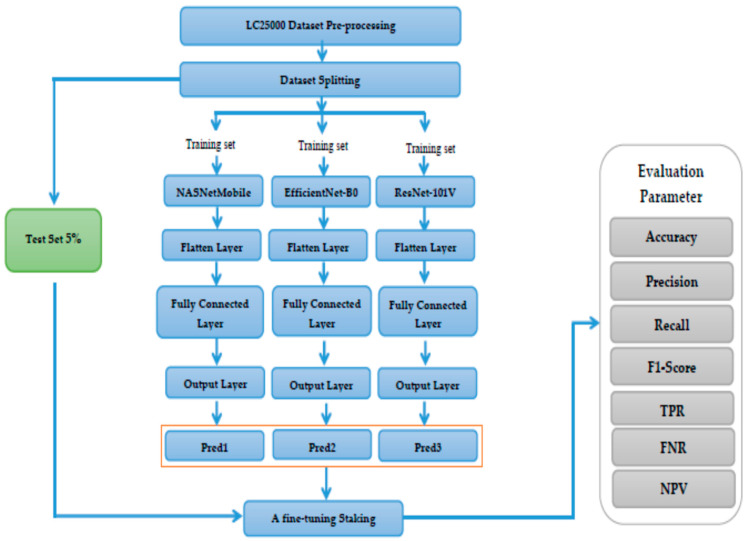
The overall model architecture.

Figure 3 depicts the overall model architecture, Algorithm 1 depicts the algorithm of the proposed model, and Algorithm 2 depicts the training algorithm of a DL mode.
**Algorithm 1:** The proposed fine-tuned DL model 1**Input** → LC25000 dataset DL
2**Output** ← Fine-tuned DL model for lung and colon prediction3**BEGIN**4      **STEP 1**: **Pre-Processing of HIs**5            **FOR EACH** image **IN** the DL
**DO**6             *Resize* HI to 224 × 2247             *Normalize* HI pixel values from [0, 255] to [0, 1]8            **END FOR**
9   **STEP 2: DL Splitting**
10         **SPLIT** DL **INTO**11             *Training se*t→ 90%
12             *Testing set* → 5%13             *Validation set* → 5%14      **STEP 3: Model Pre-Training**
15            **FOR EACH** ML IN [ResNet-101V2, NASNetMobile, EfficientNet-B0] **DO**16             *Load* and *pre-train* ML on the ImageNet dataset17             *Remove* the layer of classification from ML for feature extraction18           **END FOR**
19           **FOR EACH** pre-trained ML **DO**20             *Feed* ML with 224 × 224 × 3 HIs 21             *Feed* GlobalAveragePooling2D with the features of ML to smooth them into vectors22            **END FOR**
23           *Connect* the smoothed feature vectors from the three models into one vector24           *Add* a SoftMax layer for classification to the connected vector25      **STEP 4: Proposed Model Training and Validating**
26           **FOR I = 1 to**
N
**where**
N=5
27             **FOR EACH I DO**
28               *Fine-tune* the connected feature vector on the training set29               *Use* a validation set for early halting, according to the best model performance30               *Assess* the performance on the test set31               *Record* the test accuracies32             **END FOR**
33             *Get* the average of the recorded test accuracies34       **END FOR**
35      **STEP 5: Proposed Model Evaluation**
36           *Evaluate* the accuracy of the proposed DL model using the average test accuracies 37**END**

**Algorithm 2:** The training algorithm of a DL model 1**Input** → LC25000 dataset DL
2**Output** ← Trained model3
**BEGIN**
4      *Inputs*
→
*224 × 224 × 3*
5      *Batch_S* → 256      *Lr*
→ 0.00017      *Dropout* → 0.48      *Batch_N* →  {momentum = 0.99, epsilon = 0.001}9      *L* →{0,1,2,3,4}.10    G_A_P → Global average pooling11    *Dense* → = 412    **FOR EACH image IN DL**
13      Data_images. append(image)14    **END FOR**
15    Splitting DL16    *Model.fc* → {G_A_P, DROP, Dense}17    *Model* → Model (inputs = X.inputs, outputs = Model.fc)18    *OPT* → Adamx (0.0001)19    **FOR EACH lay in Model.layers [-20:]**
20      **IF** not instance (lay, lay. Batch_N)21                lay.trainable = True22      **END IF**
23    **END FOR**
24    Model.compile(OPT, loss = “sparse_categorical_crossentropy”).25
**END**


As depicted in Figure 3, the steps are:

**Dataset Pre-Processing.**

The first step was dataset pre-processing. This stage involved preparing the LC25000 dataset, which contains images of lung and colon cancer tissues. Pre-processing included resizing and normalization to ensure that the models receive standardized input, which helps improve their learning capabilities.

**Dataset Splitting.**

The second step was dataset splitting. The dataset was divided into a training set and a test set. The training set was used to train the models, while the test set was held out for final performance evaluation after training.

**Deep Feature Fusion.**

The third step was combining the DL models. The framework combined three pre-trained DL models: NASNetMobile, EfficientNet-B0, and ResNet-101V2. NASNetMobile was chosen to handle fine texture variations well in HI images due to its unique architecture found through neural architecture search (NAS). EfficientNet-B0’s compound scaling method makes it robust in managing both the spatial resolution and complex details of HI images, contributing to the model’s effectiveness in recognizing intricate variations in shape and texture. ResNet-101V2 is particularly adept at handling deeper architectures while learning complex features in HI images, such as the structural distinctions between cancerous and benign tissues. Each model processed the images individually, extracting different feature representations of the data.

**Model Training Process.**

The fourth step involved the model processing layers. In this step, the flattening layer converted the multi-dimensional outputs from the pre-trained models into a 1D vector, making it suitable for input into a fully connected layer. Each flattened output was fed into a fully connected layer, which learned to combine the extracted features from the pre-trained models. This layer acted as a decision-making unit for classification. The output of each model was a prediction that corresponded to the classification of the input image into one of the multiple classes.

**Fine-Tuning.**

The fifth step was the fine-tuning stage. In this step, the individual model predictions underwent fine-tuning through various techniques to improve performance. Fine-tuning allows the system to adjust the weights of each model’s contribution and combine the models’ strengths in different aspects of image classification, thereby boosting the final classification accuracy.

**Evaluation Metrics.**

The final step involved the evaluation metrics. After training, the combined model’s performance was evaluated using a series of evaluation parameters.

The combination of NASNetMobile, EfficientNet-B0, and ResNet-101V2 allowed the model to effectively learn different aspects of the HI images. NASNetMobile handled fine texture patterns, EfficientNet focused on overall structural variations, and ResNet-101V2 learned deep features.

Our architecture used three advanced models to improve the classification performance on the LC25000 dataset. The combination of models, fine-tuning, and evaluation metrics ensures the system can accurately detect different classes, including cancerous and benign tissues. This integrated approach will help to overcome the limitations of using a single model by combining the strengths of each model, leading to better accuracy and robustness in cancer detection.

#### 3.2.1. Material Pre-Processing

The pre-processing process was necessary for this research because HIs differed at the pixel level. The processed HIs had varying degrees of pixel density. Loss values at higher image values may differ from those at lower-range values. As a result, the LC25000 dataset needed to be normalized. Hence, we normalized HI pixel values from [0, 255] to [0, 1] to align the HIs and resized the HIs to 224 × 224.

#### 3.2.2. Transfer Learning

Transfer learning is an ML method where a model is trained on a source task and it is adapted for the target task. It leverages the features and knowledge gained during the training of the source model to improve the model’s performance on the target task. Transfer learning has become a common and effective approach in various fields of ML and DL due to its ability to save time, reduce data requirements, and improve the performance of models. Transfer learning allows models to perform well with fewer training data because they start with knowledge acquired from a different but related task. Training CNNs from scratch can be computationally expensive. Transfer learning saves computational resources by using pre-trained models as a starting point. Pre-trained models often capture general features, patterns, and representations useful for various tasks. These learned features can be valuable for a new task. Fine-tuning and feature extraction are the most frequently encountered forms of transfer learning. When performing feature extraction, the initial layers of a pre-existing model are employed to extract features. The extracted features are then input for a new model trained for the target task. Fine-tuning involves taking a pre-trained model and further training some or all of its layers on the target task with a new dataset. Fine-tuning allows the model to adapt to task-specific features while retaining some of the knowledge from the pre-trained model [30].

#### 3.2.3. ResNet-101V2

**Figure 4:**
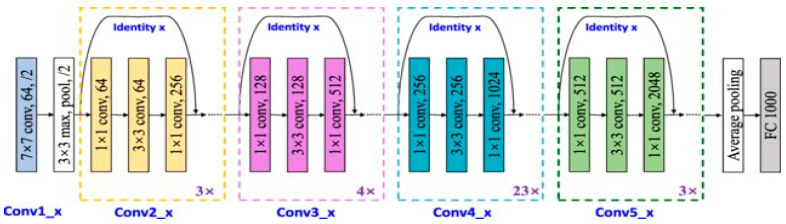
The ResNet-101V2’s architecture.

A residual network (ResNet-101V2) is a CNN with 101 layers. ResNet-101V2 is designed to solve the vanishing gradient problem. The skip connections and residual block ideas are presented in ResNet-101V2. The skip connection associates layers with additional layers by skipping a few in the middle. The skip connection is used to skip a layer if it degrades the performance. ResNets are designed by combining the residual blocks. This model is well known for its deep architecture and residual connections, which help with learning complex features over many layers. ResNet-101V2 is particularly effective at identifying fine details and preserving features across multiple levels, which is useful for detecting texture variations in HI images. ResNet-101V2 was trained on more than 1,000,000 images of the ImageNet dataset. The input images to ResNet-101V2 are 224 × 224 in size [30]. ResNet-101V2’s architecture is depicted in Figure 4.

The mathematical model of ResNet-101V2 is represented as follows: Let us denote the input to the block as X, and $F(X,\;W_{i})$ is the mapping function of the block parameterized by $W_{i}$. The output Y of the residual block is given by:

(1) $$Y=F\left(X,\;W_{i}\right)+X$$

where $W_{i}$ represents the set of parameters (weights) to be learned during training. The addition operation + is element-wise, and it is performed on the output of the mapping function and the original input. The mapping function $F\left(X,\;W_{i}\right)$ typically consists of the following operations:

1. **Batch Normalization**: Normalize the input:
  (2) $$X^{\prime }=BatchNorm\;(X)$$
2. **ReLU Activation**: Apply the rectified linear unit activation function:
  (3) $$X^{\prime \prime }=ReLU(X^{\prime })$$
3. **Convolution**: Apply a convolution operation with learnable filters:
  (4) $$F\left(X,\;W_{i}\right)=Conv\left(X^{\prime \prime },W_{i}\right)$$
4. **Another Batch Normalization**: Normalize the output of the convolution:
  (5) $$F^{\prime }\left(X,\;W_{i}\right)=BatchNorm\left(F\left(X,\;W_{i}\right)\right)$$
5. **ReLU Activation**: Apply the rectified linear unit activation function to the output of the second batch normalization:
  (6) $$F^{\prime \prime }\left(X,W_{i}\right)=ReLU\left(F^{\prime }\left(X,W_{i}\right)\right)$$
6. **Convolution**: Apply another convolution operation with learnable filters:

(7) $$F^{\prime \prime \prime }\left(X,W_{i}\right)=Conv\left(F^{\prime \prime }\left(X,W_{i}\right),W_{i}^{\prime }\right)$$

where $W_{i}^{\prime }$ represents the set of parameters for the second convolution.

Finally, the output Y is obtained by adding the output of the mapping function to the original input:

(8) $$Y=F^{\prime \prime \prime }\left(X,W_{i}\right)+X$$

#### 3.2.4. NASNetMobile

NASNetMobile [31] is a NAS network developed by researchers at Google. It utilizes a unique architecture composed of normal and reduction cells as its core functionalities. The normal cells are designed to maintain the spatial resolution of the input feature maps. This is crucial for preserving detailed information about the input data, such as subtle patterns and textures in the HIs. They focus on learning intricate patterns and feature representations at each layer while keeping the input dimensions unchanged. This allows the network to maintain fine-grained details, which is especially important for differentiating between normal and cancerous cells in HI images, where subtle differences in texture and structure are critical [32].

The reduction cells reduce the spatial dimensions of the input feature maps by down-sampling the input. This reduces the computational cost and allows the network to focus on more global features by abstracting higher-level patterns from the data. They capture higher-level, more abstract features from the input data, such as overall shape and structural variations. This is important for distinguishing large-scale features in complex images, such as differentiating between different types of tissues in colon or lung HI images. By pooling features and reducing the spatial resolution, the network can learn hierarchical feature representations, helping to manage complexity without overwhelming the model with high-dimensional data [32].

By alternating between normal and reduction cells, the NASNet architecture strikes a balance between preserving detailed, local features (via normal cells) and capturing global, abstract features (via reduction cells). This balance is crucial when dealing with high-resolution HI images that require both local texture analysis, such as detecting abnormal cell growth, and broader context recognition, such as differentiating between various tissue types.

The combination of normal and reduction cells allows NASNet to efficiently manage the complexity of the input data. For HI images, which contain both fine texture details and large structural variations, this design helps extract a wide range of relevant features. The reduction cells help prevent overfitting by reducing spatial resolution at deeper layers, while the normal cells ensure that the fine details needed for accurate classification are not lost.

**Figure 5:**
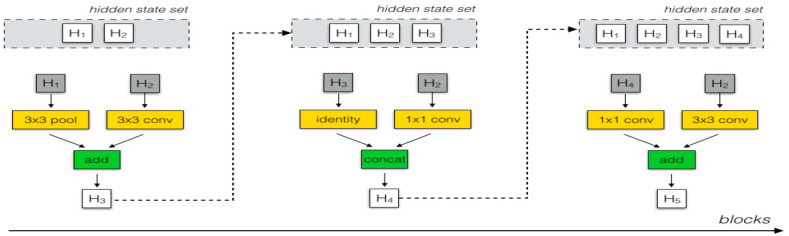
The architecture of the reduction cell and NASNet normal.

The NASNetMobile model was introduced as part of the NAS project, aiming to automatically discover the optimal network architecture for a given task. Its efficient design and ability to balance accuracy and computational requirements make it well suited for mobile applications and devices with limited resources. The NASNetMobile network is built upon reinforcement learning-optimized fundamental building cells. These cells incorporate multiple convolution, separable-convolution, and pooling operations, enhancing the model’s reliability and performance. To further improve NASNetMobile’s efficiency, a modified droppath technique called scheduled droppath is employed for effective regularization. The automated architecture search capabilities of NASNetMobile allow it to identify the best-performing network structure for specific tasks, such as image classification. This adaptability and optimization for mobile platforms has contributed to NASNetMobile’s widespread adoption and success in various applications. For texture and shape variations, NASNetMobile is particularly effective due to its flexible architecture that can capture diverse features from different image scales. Figure 5 depicts the original NASNetMobile architecture, in which reduction and normal cells are specifically utilized and the number of cells is not predetermined. The control framework within NASNetMobile can anticipate and forecast the complete network layout. To train the NASNetMobile model, around 5.3 million parameters were utilized, specifically tailored for input images that are 224 × 224 pixels in size.

The mathematical model of NASNetMobile is represented as follows: Let us denote the input to the cell as X, and $F(X,\;W_{i})$ is the mapping function of the cell parameterized by $W_{i}$. The output Y of the cell is given by:

(9) $$Y=F\left(X,\;W_{i}\right)+X$$

where $W_{i}$ represents the set of parameters (weights) to be learned during training. The addition operation + is element-wise, and it is performed on the output of the mapping function and the original input. The mapping function $F\left(X,\;W_{i}\right)$ typically consists of the following operations:

1. **Separable Convolution**: Apply a separable convolution operation:
  (10) $$F\left(X,\;W_{i}\right)=SepConv\left(X,{\;W}_{i}\right)$$
2. **Batch Normalization**: Normalize the output of the separable convolution:
  (11) $$X^{\prime }=BatchNorm\;(X)$$
3. **Convolution**: Apply a convolution operation with learnable filters:
  (12) $$F^{\prime }\left(X,\;W_{i}\right)=BatchNorm\left(F\left(X,\;W_{i}\right)\right)$$
4. **ReLU Activation**: Apply the rectified linear unit activation function to the output of the batch normalization:
  (13) $$F^{\prime \prime }\left(X,{\;W}_{i}\right)=ReLU\left(F^{\prime }\left(X,W_{i}\right)\right)$$
5. **Pooling:** Apply the pooling operation:
  (14) $$F^{\prime \prime \prime }\left(X,{\;W}_{i}\right)=Pooling\left(F^{\prime \prime }\left(X,W_{i}\right)\right)$$

Finally, the output Y is obtained by adding the output of the mapping function to the original input:

(15) $$Y=F^{\prime \prime \prime }\left(X,W_{i}\right)+X$$

#### 3.2.5. EfficientNet-B0

EfficientNet is a compound scaling method that scales network width, length, and resolution using a group of fixed coefficients. Scaling width increases feature maps in each layer. Scaling depth increases the layers of the network. Scaling resolution increases the resolution of input images [33]. Tan and Le [33] proposed seven models, ranging from EfficientNet-B0 to EfficientNet-B7. When applied to the ImageNet dataset, EfficientNet CNN models demonstrated superior performance in terms of parameters and the number of Top-1 accuracy [34].

The most crucial part of the EfficientNet model family is the mobile inverted bottleneck convolution (MBConv). MBConv was developed based on the concepts of the MobileNet models [35]. One of the main ideas is the use of depth-wise separable convolutions, which necessitates a depth-wise convolution and a layering pointwise. The two additional ideas were born out of MobileNet-V2: linear bottlenecks and inverted residual connections.

EfficientNet-B0 aims to achieve improved classification accuracy while using fewer computational resources. It accomplishes this by scaling the depth, width, and resolution of the network in a balanced manner. The compound scaling approach of EfficientNet-B0 helps capture both large-scale and fine-grained features related to shape and texture, making it well suited for the task of HI image classification.

EfficientNet-B1 is the first scaled version, with roughly twice as much capacity. There are 237 layers in EfficientNet-B0 and 813 layers in EfficientNet-B7.

**Figure 6:**
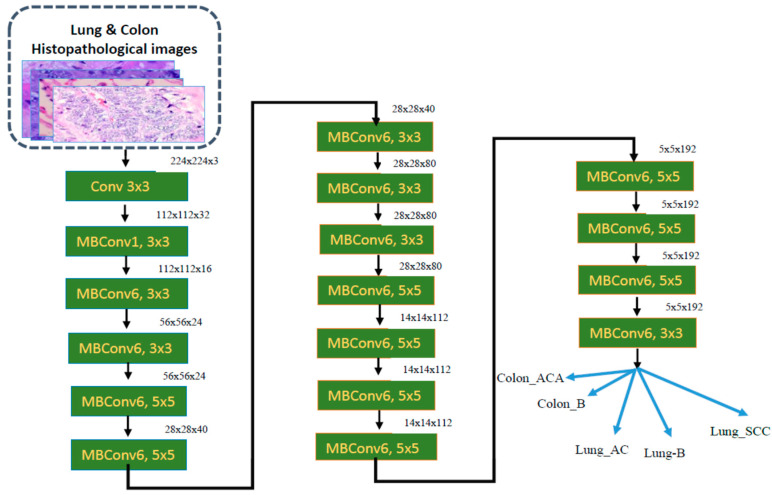
The architecture of EfficientNet-B0.

Table 2 displays the specifics of the scale for the models EfficientNet-B0, and Figure 6 shows the architecture of EfficientNet-B0 [36].

The mathematical model of EfficientNet-B0 is represented as follows: Let us denote the input to the block as X, and $F(X,\;W_{i})$ is the mapping function of the block parameterized by $W_{i}$. The output Y of the block is given by:

(16) $$Y=F\left(X,\;W_{i}\right)+X$$

where $W_{i}$ represents the set of parameters (weights) to be learned during training. The addition operation + is element-wise, and it is performed on the output of the mapping function and the original input. The mapping function $F\left(X,\;W_{i}\right)$ typically consists of the following operations:

1. **Depth-wise Separable Convolution**: Apply a depth-wise separable convolution operation:
  (17) $$F\left(X,\;W_{i}\right)=DepthwiseSepConv\;\left(X,\;W_{i}\right)$$
2. **Batch Normalization**: Normalize the output of the depth-wise separable convolution:
  (18) $$F^{\prime }\left(X,\;W_{i}\right)=BatchNorm\left(F\left(X,\;W_{i}\right)\right)$$
3. **Swish Activation**: Apply the swish activation function to the output of batch normalization:
  (19) $$F^{\prime \prime }\left(X,W_{i}\right)=Swich\left(F^{\prime }\left(X,W_{i}\right)\right)$$
4. **Squeeze-and-Excitation**: Apply the squeeze-and-excitation operation to capture channel-wise dependencies:
  (20) $$F^{\prime \prime \prime }\left(X,{\;W}_{i}\right)=SE\left(F^{\prime \prime }\left(X,W_{i}\right)\right)$$
5. **Linear Projection**: Project the features using a linear projection:
  (21) $$F^{\prime \prime \prime }\left(X,W_{i}\right)=LinerProjection\left(F^{\prime \prime \prime }\left(X,W_{i}\right)\right)$$

Finally, the output Y is obtained by adding the output of the mapping function to the original input:

(22) $$Y=F^{\prime \prime \prime }\left(X,W_{i}\right)+X$$

#### 3.2.6. Integration of CNN Features

Integrating features from CNNs means combining the strengths of various CNN designs to enhance the performance of a DL model. This process, called integrative feature fusion, can result in better accuracy, generalization, and robustness. Each CNN extracts features from the input data, capturing different levels of abstraction. These features range from low-level details like edges and textures to high-level semantic information. By merging the features extracted from different CNNs, we create a richer and more comprehensive representation of the data. A new model architecture is then developed to leverage these fused features, often leading to improved performance [37].

In our model, we used three DL models: ResNet-101V2, NASNetMobile, and EfficientNet-B0. To handle the texture complexities in HI images, the combination of ResNet-101V2 and EfficientNet-B0 played a crucial role. ResNet-101V2’s depth and ability to extract detailed patterns across many layers helped with recognizing fine-grained textures, while EfficientNet-B0’s efficient scaling enabled the model to generalize well across varying texture resolutions. NASNetMobile, with its ability to dynamically adjust its architecture during training, allowed the model to better capture variations in the shape of HI structures. This, combined with the hierarchical feature extraction of ResNet-101V2, made the ensemble capable of distinguishing between different shape variations more effective.

The outputs from the intermediate layers of each model were extracted and fused to create a more robust representation of the image. This ensured that the model benefitted from the strengths of each architecture: ResNet-101V2’s detailed texture extraction, NASNetMobile’s adaptable architecture, and EfficientNet-B0’s balanced feature scaling. Attention layers were added after the feature fusion to ensure the model focused on the most important regions in the images, thus improving performance when texture and shape variations were subtle. After the integrative feature extraction, a custom dense classifier was used to better distinguish between the classes. The classifier was fine-tuned using techniques like dropout and batch normalization to ensure it generalized well across different classes. The integrative approach led to a noticeable improvement in multiclassification accuracy. By fusing features from diverse architectures, the model captured a richer set of representations, addressing the issues of texture and shape variations more effectively than individual models. Additionally, the use of attention mechanisms and custom classifiers further boosted performance, helping the model better discriminate between different classes. This approach not only enhanced the model’s ability to handle the complex variations in HI images but also raised the overall classification accuracy, making it a more robust solution for detecting lung and colon cancer.

In our implementation, we initially used the ImageNet dataset, which has more than millions of images, to pre-train the three CNN models. Each model had an input of images of 244 × 244 × 3 in size. ResNet-101V2 extracted 2048 features, NASNetMobile extracted 1956 features, and EfficientNet-B0 extracted 1280 features. After the pre-training phase, the pre-trained models used the GlobalAveragePooling2D simultaneously to smooth all layers into a feature vector by obtaining the average for each input channel. Moreover, a concatenate layer was used to fuse the pre-trained individual feature vectors of ResNet-101V2, NASNetMobile, and EfficientNet-B0 into a single feature vector. In addition, the fusion of a single feature vector was fine-tuned on the training set of the LC25000 dataset. In the fine-tuning phase, we fine-tuned the concatenated single feature vector on the test set of the LC25000 dataset by using six layers (dropout, batch_normalization, dense, dropout_1, batch_normalization_1, and dense_1). Since the LC25000 dataset has five classes, the last dense layer had five neurons. Table 3 shows the structure of the proposed fine-tuned DL model.

## 4. Model Implementation and Evaluation

### 4.1. Measured Metrics

The LC25000 dataset was utilized to measure our proposed model’s specificity, accuracy, FNR, NPV, precision, recall, and F1-score, as mentioned in Equations (23)–(29):

(23) $$Accuracy=\frac{\left(TP+TN\right)}{\left(TP+FP+TN+FN\right)}$$

(24) $$Precision=\frac{TP}{\left(TP+FP\right)}$$

(25) $$Sensivity=\frac{TP}{\left(TP+FN\right)}$$

(26) $$Specifity=\frac{TN}{\left(TN+FP\right)}$$

(27) $$F1-score=\frac{2\times Precision\times Recall}{Precision+Recall}$$

(28) $$False\;negative\;rate\;(FNR)=\frac{FN}{TP+FN}$$

(29) $$Negative\;predictive\;value\;(NPV)=\frac{TN}{TN+FN}$$

NPV means the ratio of TN predictions that are normal. FNR means the ratio of FN. TP + FN is the sum of the patients who had tumors. True positive (TP) indicates a positive real value and a positive classification. False negative (FN) indicates that the actual value is positive even though it is categorized as negative. False positive (FP) means that the true value is negative when it is classified positively. True negative (TN) indicates a negative real value and a negative classification.

The accuracy is the percentage of the number of correct predictions out of the total number of predictions. Precision is the ratio of true positive predictions to the total number of positive predictions (both true positives and false positives). The proportion of actual cases with positive outcomes that were predicted to be positive is called sensitivity. The proportion of predicted negatives that occurred is known as specificity. The harmonic mean of precision and sensitivity is the F1-score.

### 4.2. The Proposed Fine-Tuned DL Model Implementation

In this research, we implemented the proposed fine-tuned ensemble model using the Kaggle platform. Due to time constraints during the study, we did not employ formal hyperparameter optimization methods, such as grid search or Bayesian optimization. Instead, we opted for standard default values based on their established reliability in the literature—specifically, learning rate, dropout rate, and batch size. The values of 0.001 for the learning rate, 0.4 for the dropout rate, and 25 for the batch size were selected, as they are commonly used in similar models and tasks. These defaults were chosen to ensure stable model performance without requiring extensive tuning. The hyperparameter values used in the implementation are shown in Table 4.

### 4.3. The Proposed DL Model Evaluation

ResNet-101V2, NASNetMobile, EfficientNet-B0, and the proposed fine-tuned DL model were evaluated over the LC25000 dataset. It was separated into a 90% training set with 11,250 HIs, a 5% test set with 625 HIs, and a 5% validation set with 625 HIs.

We applied the transfer learning process that pre-trained the three CNN models on the ImageNet dataset. After the supervised pre-training phase, they used GlobalAveragePooling2D simultaneously to smooth all layers into a feature vector by obtaining the average for each input channel. A concatenate layer was used to fuse the pre-trained individual feature vectors of ResNet-101V2, NASNetMobile, and EfficientNet-B0 into a single feature vector. In addition, the fusion single feature vector was fine-tuned on the training set of the LC25000 dataset. Finally, the evaluated metrics in Equations (23)–(29) were applied to ResNet-101V2, NASNetMobile, EfficientNet-B0, and the proposed fine-tuned DL model.

We implemented five experiments for the multi-classification of the LC25000 dataset using ResNet101, NASNetMobile, EfficientNet-B0, the proposed fusion DL model, and the stacked ensemble model.

In the first three experiments, ResNet101, NASNetMobile, and EfficientNet-B0 were pre-trained over the ImageNet dataset and fine-tuned on the training set of the LC25000 dataset to fine-tune their parameters. They used GlobalAveragePooling2D simultaneously to smooth all layers into a feature vector by obtaining the average value for each input channel. Finally, each CNN model was used for the multi-classification and measured by the measured metrics.

In the fourth experiment, we used the stacking algorithm to stack ResNet-101V2, NASNetMobile, and EfficientNet-B0 based on the majority voting technique. The stacked ensemble model was used for the multi-classification and evaluated by the evaluation metrics.

In the five experiments, we combined each CNN model’s last fully connected layers, fine-tuned the fusion single feature vector on the training set of the LC25000 dataset, and evaluated it by the measured metrics.

Table 5, Table 6, Table 7, Table 8 and Table 9 demonstrate the multi-classification outcomes of the five experiments on the test set of the LC25000 using the proposed fusion DL model, the stacked ensemble model, EfficientNet-B0, NASNetMobile, and ResNet-101V2, respectively. The five experiments classified the five tissue classes of the LC25000 dataset: benign lung tissues, adenocarcinoma, squamous cell carcinoma, benign colonic tissues, and colon adenocarcinoma.

Table 5, Table 6, Table 7, Table 8 and Table 9 demonstrate the average measured metrics for the proposed fusion DL model, the stacked ensemble model, EfficientNet-B0, NASNetMobile, and ResNet-101V2, respectively. The average accuracy was 99.94%, 99.55%, 97.18%, 93.66%, and 99.74%, respectively, for the proposed fusion DL model, the stacked ensemble model, EfficientNet-B0, NASNetMobile, and ResNet-101V2. **Hence, the fusion DL model had the best results, with an accuracy of 99.94%.**

The proposed fusion model recorded averages of 99.96%, 0.16%, 99.96%, 99.84%, 99.84%, and 99.84% for specificity, FNR, NPV, precision, recall, and F1-score, respectively. The average specificity, FNR, NPV, precision, recall, and F1-score for the stacked ensemble model were 99.20%, 1.60%, 99.60%, 96.85%, 98.40%, and 97.62%, respectively. EfficientNet-B0 yielded 98.24%, 6.68%, 98.35%, 94.65%, 92.96%, and 92.80% for the specificity, FNR, NPV, precision, recall, and F1-score averages, respectively. NASNetMobile achieved 95.99%, 16.34%, 96.36%, 89.32%, 84.16%, and 83.72% for the specificity, FNR, NPV, precision, recall, and F1-score averages, respectively. ResNet-101V2 recorded 99.84%, 0.62%, 99.84%, 99.36%, 99.36%, and 99.36% for the specificity, FNR, NPV, precision, recall, and F1-score averages, respectively.

Therefore, the proposed fusion DL model recorded the highest averages of specificity, NPV, precision, recall, and F1-score, at 99.96%, 99.96%, 99.84%, 99.84%, and 99.84%, respectively, and it achieved the lowest FNR, at 0.16%. NASNetMobile achieved the highest FNR, at 16.34%. Hence, **the proposed fusion DL model performed well, with relatively high values for most of the evaluation criteria, making it a strong performer for the multi-classification task of lung and colon tumors**.

Table 5, Table 6, Table 7, Table 8 and Table 9 provide an in-depth look at the multi-classification process’s outcomes. The LC25000 was divided into five categories through this procedure: Colon_Adenocarcinoma (Colon_ACA), Colon_Benign_Tissue (Colon_B_T), Lung-Benign_Tissue (Lung_B_T), Lung_Adenocarcinoma (Lung_ACA), and Lung_Squamous_Cell_Carcinoma (Lung_SCC). Over the LC25000 dataset, the proposed fusion DL model, the stacked ensemble model, EfficientNet-B0, NASNetMobile, and ResNet-101V2 were evaluated through the calculations of the accuracy, precision, specificity, recall, FNR, NPV, and F1-score for each class.

For the **Colon_Adenocarcinoma** class, the accuracy, precision, specificity, recall, NPV, and F1-score were 100% for the proposed fusion model, the stacked ensemble model, EfficientNet-B0, and ResNet-101V2, and they achieved 0% for FNR.

For the **Colon_Benign_Tissue** class, the accuracy, precision, specificity, recall, NPV, and F1-score were 100% for the proposed fusion model, the stacked ensemble model, EfficientNet-B0, NASNetMobile, and ResNet-101V2, and they achieved 0% for FNR.

For the **Lung_Adenocarcinoma** class, the proposed fusion model achieved the highest accuracy, specificity, NPV, precision, recall, and F1-score at 99.84%, 99.80%, 100%, 99.25%, 100%, and 99.62%, respectively, and it recorded the lowest FNR at 0%. Moreover, EfficientNet-B0 had the highest specificity at 99.80%.

For the **Lung-Benign_Tissue** class, the proposed fusion model and ResNet-101V2 achieved the highest accuracy, specificity, NPV, precision, recall, and F1-score at 100%, and it recorded the lowest FNR at 0%. In addition, the proposed stacked ensemble model, EfficientNet-B0 and NASNetMobile, had the highest specificity and precision at 100%.

For the **Lung_Squamous_Cell_Carcinoma** class, the proposed fusion recorded the highest precision, specificity, accuracy, and F1-score at 100%, 100%, 99.84%, and 99.60%, respectively. EfficientNet-B0 had the highest NPV and recall at 100% and the lowest FNR at 0%.

**Figure 7:**
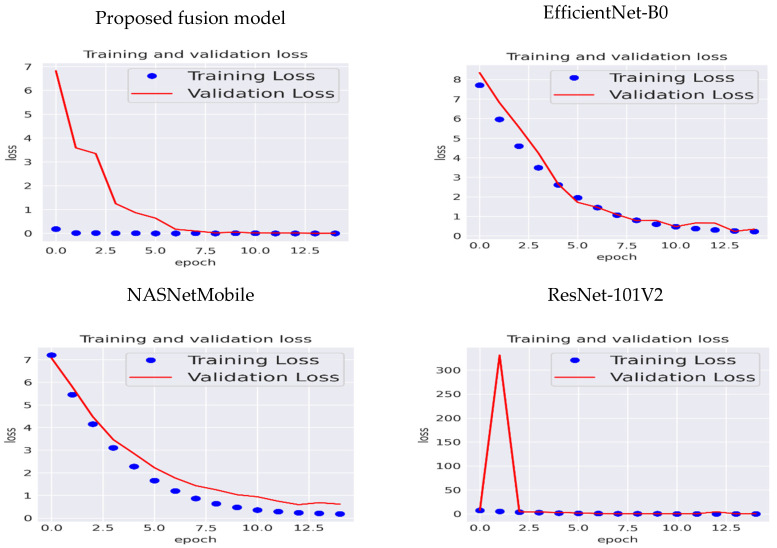
Training and validation loss of the three CNN models and the proposed fusion model.

The training and validation loss of the proposed fusion DL model, EfficientNet-B0, NASNetMobile, and ResNet-101V2, is depicted in Figure 7. For the proposed fusion DL model, the training loss began at a very low value and remained consistently low throughout all training epochs. This suggests that the model quickly minimized the loss on the training data and maintained a very low error, indicating a good fit to the training data. The validation loss started at a significantly high value (approximately 7) and decreased sharply during the initial few epochs. After about five epochs, the validation loss stabilized and remained very low. The rapid drop in validation loss followed by stabilization suggests that the model quickly learned to generalize well on unseen validation data. There was no evidence of overfitting, as both training and validation losses were low and aligned after the initial few epochs. Moreover, the model efficiently reduced both training and validation losses early in the training process, indicating that it generalized well to new data after only a few epochs.

For EfficientNet-B0 and NASNetMobile, the training and validation loss were very close and decreased as the number of epochs increased. Hence, they were not biased or variances. For the ResNet-101V2, at the beginning of the experiment, the validation loss was very high, but after the second epoch, it decreased and equaled 0%, similar to the training loss. Hence, it was not biased and displayed no variance.

**Figure 8:**
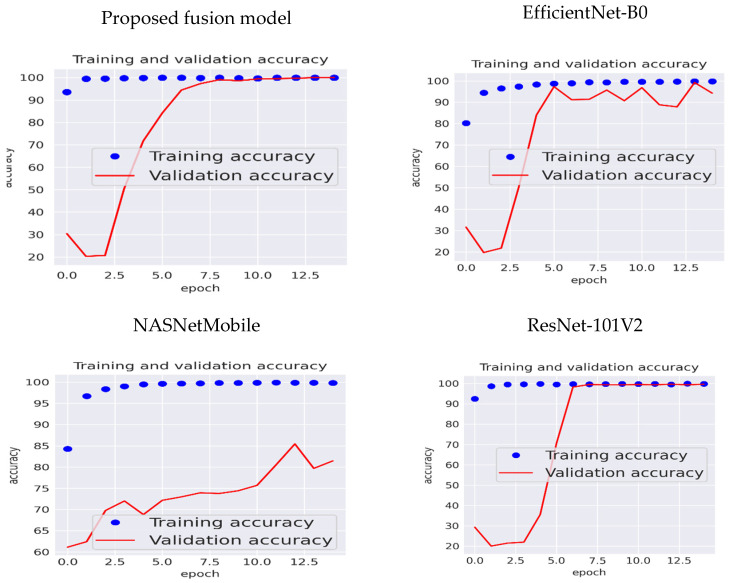
Training and validation accuracy of the three CNN models and the proposed fusion model.

Figure 8 depicts the validation and training accuracy of the proposed model, Effi-cientNet-B0, NASNetMobile, and ResNet-101V2. The training accuracy for the proposed fusion model was 100% after the 2nd epoch, and the validation accuracy was 100% after the 10th epoch. For the EfficientNet-B0 model, the training accuracy was 100% after the fifth epoch, and the validation accuracy was between 90% and 100% after the fifth epoch. For the NASNetMobile model, the training accuracy was 100% after the fifth epoch, and the maximum validation accuracy was 85%. ResNet-101V2’s training and validation accuracy was 100% after the fifth epoch.

The observed rapid convergence, where the proposed model achieved 100% training accuracy by the second epoch, can be attributed to the ensemble of three highly effective architectures: NASNetMobile, EfficientNet-B0, and ResNet-101V2. Each of these models brings distinct strengths in feature extraction. NASNetMobile is known for its ability to balance accuracy and computational efficiency. EfficientNet-B0 scales efficiently with minimal computational resources while still maintaining accuracy. ResNet-101V2 is particularly effective at preserving feature information over deeper layers, preventing vanishing gradients.

This fusion model accelerated the learning process by efficiently capturing diverse features and reducing the time required for the classifier to optimize. The complementary nature of these models enabled quick convergence while maintaining robust performance across training and validation sets. Furthermore, to ensure robust generalization, we extended the analysis to include precision, recall, F1-score, and confusion matrix evaluations. These metrics, alongside validation accuracy, demonstrated that the model maintained high performance on unseen data, further confirming that the rapid training convergence did not lead to overfitting.

For the EfficientNet-B0 model, the training accuracy was 100% after the fifth epoch, and the validation accuracy was between 90% and 100% after the fifth epoch. For the NASNetMobile model, the training accuracy was 100% after the fifth epoch, and the maximum validation accuracy was 85%. ResNet-101V2’s training and validation accuracy was 100% after the fifth epoch.

**Figure 9:**
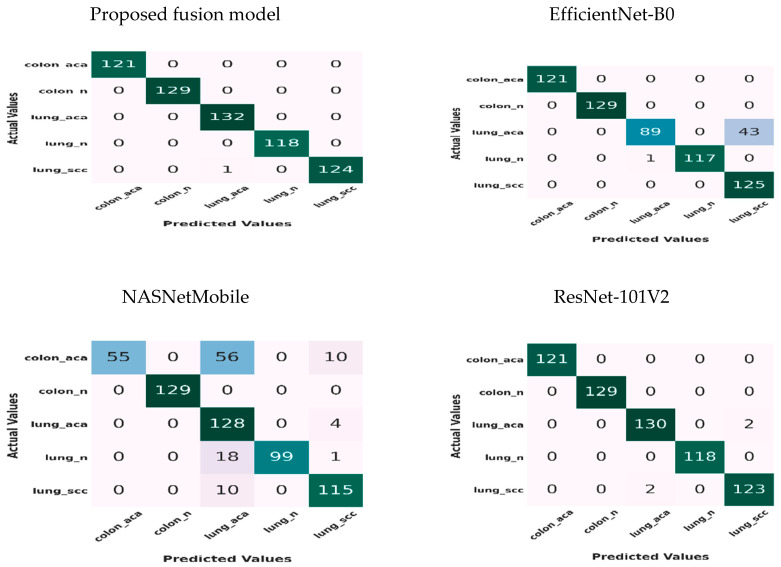
The confusion matrix for the three CNN models and the proposed fusion model on the test set.

Figure 9 depicts the confusion matrix for the proposed fusion model, EfficientNet-B0, NASNetMobile, and ResNet-101V2. There were five classes of the test set of the LC25000 dataset: Colon_Adenocarcinoma (121 HIs), Colon_Benign_Tissue (129 HIs), Lung-Benign_Tissue (118 HIs), Lung_Adenocarcinoma (132 HIs), and Lung_Squamous_Cell_Carcinoma (125 HIs).

For the Colon_Adenocarcinoma class, the proposed fusion model, stacked ensemble model, EfficientNet-B0, and ResNe-t101 classified 121 of 121 HIs and achieved an accuracy of 100%. NASNetMobile classified 55 of 121 HIs and achieved an accuracy of 45.4%. Hence, the proposed fusion model, the stacked ensemble model, EfficientNet-B0, and ResNet-101V2 had the best performance and followed NASNetMobile.

For the Colon_Benign_Tissue class, the accuracy of the proposed fusion model, the stacked ensemble model, EfficientNet-B0, NASNetMobile, and ResNet-101V2 was 100%, as they classified 129 of 129 HIs.

For the Lung_Adenocarcinoma class, the proposed fusion model classified 132 of 132 HIs and recorded an accuracy of 100%. The stacked ensemble and NASNetMobile models classified 128 of 132 HIs and achieved an accuracy of 96.9%. EfficientNet-B0 predicted 89 of 132 HIs and recorded an accuracy of 67.4%. ResNet-101V2 predicted 130 of 132 HIs and achieved an accuracy of 98.4%. Hence, the proposed fusion model performed best, followed by ResNet-101V2, the stacked ensemble, NASNetMobile, and EfficientNet-B0.

For the Lung-Benign_Tissue class, the proposed fusion model and ResNet-101V2 classified 118 of 118 HIs and recorded an accuracy of 100%. The stacked ensemble and EfficientNet-B0 models classified 117 of 118 HIs and achieved an accuracy of 99.1%. NASNetMobile classified 99 of 118 HIs and recorded an accuracy of 83.8%. Hence, the proposed fusion model and ResNet-101V2 recorded the best performance, followed by the stacked ensemble, the EfficientNet-B0 model, and NASNetMobile.

For the Lung_Squamous_Cell_Carcinoma class, the proposed fusion model classified 124 of 125 HIs and recorded an accuracy of 99.2%. The stacked ensemble and ResNet-101V2 classified 123 of 125 HIs and achieved an accuracy of 98.4%. EfficientNet-B0 classified 125 of 125 HIs and recorded an accuracy of 100%. NASNetMobile classified 115 of 125 HIs and recorded an accuracy of 92%. Hence, EfficientNet-B0 recorded the best performance, followed by the proposed fusion model, the stacked ensemble, ResNet-101V2, and NASNetMobile.

### 4.4. Model Result Comparison with the Literature

Our proposed model based on feature fusion outperformed the most recent research, displayed in Table 10. The accuracy of the proposed fine-tuned DL model recorded was 99.94%. From the patient’s point of view, the proposed fine-tuned model will reduce the cost and time for colon and lung diagnosis, hence providing appropriate treatment. Compared to a stacked ensemble of the same base learners and other recent studies, the assessment of the proposed fusion model uncovered that, with tuning, it is an unmatched discerning outcome.

## 5. Conclusions

We proposed a fine-tuned fusion DL model of EfficientNet-B0, NASNetMobile, and ResNet-101V2 for the multi-classification task of colon and lung cancers. The proposed fine-tuned model was based on feature fusion. The proposed fine-tuned model will assist pathologists in detecting lung and colon cancer early with less effort, time, and expense. Each of EfficientNet-B0, NASNetMobile, and ResNet-101V2 needs to be improved regarding varieties in the texture and shape of the input images. However, the proposed fusion model recorded a high accuracy in the multi-classification task of lung and colon cancer since it joined the benefits of the three CNN models to increase the accuracy of the results. We pre-trained the three CNN models on the ImageNet dataset. The three pre-trained models used a GlobalAveragePooling2D method to smooth all layers into a vector. The GlobalAveragePooling2D method computes the average of each feature map across its whole spatial extent, providing a single value per feature map. We combined the pre-trained individual vectors of the pre-trained EfficientNet-B0, NASNetMobile, and ResNet-101V2 models into a single vector by a concatenate layer. The concatenated single feature vector was fine-tuned on the training set of the LC25000 dataset. We evaluated the proposed model over the LC25000 dataset, which includes colon and lung HIs. The LC25000 dataset was pre-processed by normalization and resizing techniques. The LC25000 dataset was split into a training set (90%), a test set (5%), and a validation set (5%). The proposed fine-tuned model was measured and compared with the stacked ensemble and recent DL classifies. The proposed fusion model recorded 99.84%, 99.84%, 99.84%, 99.94%, 99.96%, 99.84%, 0.16%, and 99.96%, for precision, recall, F1-score, accuracy, specificity, FNR, and NPV, respectively. Therefore, the proposed fine-tuned fusion model delivered cutting-edge performance across all classes. The current limitation of our model is the processing speed, which poses a challenge for our developing system. In future research, we plan to explore the proposed model on a variety of human cancer types and employ hyperoptimization algorithms to automatically enhance the hyperparameterization process.

## Figures and Tables

**Table 1:** The features of the five classes of the LC25000 dataset.

Class	Features
NSCLC	Large and irregular nuclei. Atypical cells with varying shapes and sizes. Hyperchromatic and prominent nucleoli. Disorganized tissue structure and evident nuclear pleomorphism.
SCLC	Small, round to oval cells with scant cytoplasm. Finely granular chromatin, often called “salt-and-pepper” chromatin. High nuclear-to-cytoplasmic ratio. Dense clusters of cancer cells with frequent mitosis and necrosis.
Benign lung tissue	Well-organized structure of lung tissue with clearly defined alveoli. Normal-sized nuclei without irregularities. Lack of abnormal cell growth, typical cell architecture, and absence of mitosis. Regular shapes and sizes of the cells.
Colon cancer tissue	Disorganized and distorted glandular structures. Abnormal nuclear shapes and sizes with prominent nucleoli. High cellular density and possible necrotic areas. Loss of normal tissue architecture, with cells invading the surrounding stroma.
Benign colon tissue	Well-organized glandular structures and epithelial cells. Uniform nuclei and cells with no signs of atypia. No invasion into surrounding tissues, and normal tissue architecture is intact. Lack of inflammatory or necrotic changes.

**Table 2:** EfficientNet-B0.

Model Name	W Coeff.	D. Coeff.	Resolution	Dropout Rate
EfficientNet-B0	1.0	1.0	224	0.2

**Table 3:** Structure of the proposed fine-tuned DL model.

Layer	Input	Output
InputLayer	(None,224,224,3)	(None,224,224,3)
EfficientNet-B0	(None,224,224,3)	(None,7,7,1280)
NASNetMobile	(None,224,224,3)	(None,7,7,1056)
ResNet-101V2	(None,224,224,3)	(None,7,7,2048)
GlobalAveragePooling2D	(None,7,7,1280)	(None,1280)
GlobalAveragePooling2D	(None,7,7,1056)	(None,1056)
GlobalAveragePooling2D	(None,7,7,2048)	(None,2048)
Concatenate	(None,1280), (None,1056), (None,2048)	(None,4384)
Dropout	(None,4384)	(None,4384)
BatchNormailzation	(None,4384)	(None,4384)
Dense	(None,4384)	(None,128)
Dropout	(None,128)	(None,128)
BatchNormailzation	(None,128)	(None,128)
Dense	(None,128)	(None,5)

**Table 4:** The values of the hyperparameters.

Parameter	Value
img_size	224 × 224
Number of epochs	15
Batch size	25
Activation	Softmax
Optimizer	Adam
Initial learning rate	1 × 10^−3^
Decay	0.00001
Factor to reduce LR	0.5
Dropout	0.4

**Table 5:** The proposed fusion results from the multi-classification.

Class	Accuracy (%)	Specificity (%)	FNR (%)	NPV (%)	Precision (%)	Recall (%)	F1-Score (%)
Colon_ACA	100	100	0	100	100	100	100
Colon_B_T	100	100	0	100	100	100	100
Lung_ACA	99.84	99.80	0	100	99.25	100	99.62
Lung_B_T	100	100	0	100	100	100	100
Lung_SCC	99.84	100	0.80	99.80	100	99.20	99.60
**Average**	**99.94**	**99.96**	**0.16**	**99.96**	**99.84**	**99.84**	**99.84**

**Table 6:** The stacked ensemble model results from the multi-classification process.

Class	Accuracy (%)	Specificity (%)	FNR (%)	NPV (%)	Precision (%)	Recall (%)	F1-Score (%)
Colon_ACA	100	100	0	100	100	100	100
Colon_B	100	100	0	100	100	100	100
Lung_ACA	98.88	99.39	3.03	99.19	97.71	96.97	97.34
Lung-B	99.84	100	0.85	99.80	100	99.15	99.57
Lung_SCC	99.04	99.20	1.60	99.60	96.85	98.40	97.62
**Average**	**99.55**	**99.72**	**1.10**	**99.72**	**98.89**	**98.88**	**98.88**

**Table 7:** The results of EfficientNet-B0 for the multi-classification process.

Class	Accuracy (%)	Specificity (%)	FNR (%)	NPV (%)	Precision (%)	Recall (%)	F1-Score (%)
Colon_ACA	100	100	0	100	100	100	100
Colon_B	100	100	0	100	100	100	100
Lung_ACA	92.96	99.80	32.58	91.96	98.89	67.42	80.18
Lung-B	99.84	100.00	0.85	99.80	100.00	99.15	99.57
Lung_SCC	93.12	91.40	0.00	100.00	74.40	100.00	85.32
**Average**	**97.18**	**98.24**	**6.68**	**98.35**	**94.65**	**92.96**	**92.80**

**Table 8:** The results of NASNetMobile for the multi-classification process.

Class	Accuracy (%)	Specificity (%)	FNR (%)	NPV (%)	Precision (%)	Recall (%)	F1-Score (%)
Colon_ACA	89.44	100	54.55	88.42	100	45.45	62.50
Colon_B	100	100	0	100	100	100	100
Lung_ACA	85.92	82.96	3.03	99.03	60.38	96.97	74.42
Lung-B	96.96	100.00	16.10	96.39	100.00	83.90	91.24
Lung_SCC	96.00	97.00	8	97.98	88.46	92.00	90.20
**Average**	**93.66**	**95.99**	**16.34**	**96.36**	**89.32**	**84.16**	**83.72**

**Table 9:** The results of ResNet-101V2 for the multi-classification process.

Class	Accuracy (%)	Specificity (%)	FNR (%)	NPV (%)	Precision (%)	Recall (%)	F1-Score (%)
Colon_ACA	100	100	0	100	100	100	100
Colon_B	100	100	0	100	100	100	100
Lung_ACA	99.36	99.59	1.52	99.59	98.48	98.48	98.48
Lung-B	100	100	0.00	100	100	100	100
Lung_SCC	99.36	99.60	1.60	99.60	98.40	98.40	98.40
**Average**	**99.74**	**99.84**	**0.62**	**99.84**	**99.36**	**99.36**	**99.36**

**Table 10:** Comparing the proposed fine-tuned technique and recent research.

Reference	Methodology	Accuracy	Dataset
A. H. Chehade1 et al. [1]	XGBoost, SVM, RF, LDA, and MLP	99%	LC25000
M. Ali and R. Ali [20]	CNNs	99.58%	LC25000
Sakr, A.S. et al. [21]	CNN	99.50%	LC25000
M. Masud et al. [22]	CNN	96.33%	LC25000
Naresh Kumar et al. [23]	RF and deep features from DenseNet-121	98.60%	LC25000
M. Shahid et al. [24]	AlexNet	98.4%	LC25000
Md. A. Talukder et al. [25]	VGG16, VGG1, MobileNet, DenseNet169, DenseNet201, Ensemble, and feature extraction	99.05%	LC25000
O. Attallah et al. [26]	MobileNet, SqueezeNet, ShuffleNet, PCA, fast Walsh–Hadamard, and discrete wavelet	99.6%	LC25000
J. D. Akinyemi et al. [27]	EfficientNet-B7	99.72%.	LC25000
**The stacked ensemble model**	**EfficientNet-B0, NASNetMobile, and ResNet-101V2.**	**99.55%**	**LC25000**
**The proposed model**	**Fine-tuned model based on feature fusion**	**99.94%**	**LC25000**

## Data Availability

The dataset referenced in this article is the LC25000 dataset, obtained from https://opendatalab.com/OpenDataLab/LC25000 accessed on 21 August 2024. This dataset was issued on 2019 and it is available at no cost to all researchers and scientists for the purpose of conducting experiments.

## References

[1] Siegel R.L., Miller K.D., Jemal A. (2020). Cancer statistics. CA Cancer J. Clin..

[2] WHO Cancer. https://www.who.int/news-room/fact-sheets/detail/cancer.

[3] Chehade A.H., Abdallah N., Marion J.-M., Oueidat M., Chauvet P. (2022). Lung and colon cancer classification using medical imaging: A feature engineering approach. Phys. Eng. Sci. Med..

[4] Togaçar M. (2021). Disease type detection in lung and colon cancer images using the complement approach of inefficient sets. Comput. Biol. Med..

[5] Bermúdez A., Arranz-Salas I., Mercado S., López-Villodres J.A., González V., Ríus F., Ortega M.V., Alba C., Hierro I., Bermúdez D. (2021). Her2-positive and microsatellite instability status in gastric cancer—Clinicopathological implications. Diagnostics.

[6] Zhu D., Ding R., Ma Y., Chen Z., Shi X., He P. (2021). Comorbidity in lung cancer patients and its association with hospital readmission and fatality in China. BMC Cancer.

[7] Schüz J., Espina C. (2021). The eleventh hour to enforce rigorous primary cancer prevention. Mol. Oncol..

[8] Raman S. (2020). Can curcumin along with chemotherapeutic drugs and lipid provide an effective treatment of metastatic colon cancer and alter multidrug resistance?. Med. Hypotheses.

[9] Bergers G., Fendt S.M. (2021). The metabolism of cancer cells during metastasis. Nat. Rev. Cancer.

[10] Koo M.M., Swann R., McPhail S., Abel G.A., Elliss-Brookes L., Rubin G.P., Lyratzopoulos G. (2020). Presenting symptoms of cancer and stage at diagnosis: Evidence from a cross-sectional, population-based study. Lancet Oncol..

[11] Zhou W., Liu G., Hung R.J., Haycock P.C., Aldrich M.C., Andrew A.S., Arnold S.M., Bickeböller H., Bojesen S.E., Brennan P. (2021). Causal relationships between body mass index, smoking, and lung cancer: Univariable and multivariable Mendelian randomization. Int. J. Cancer.

[12] Khan T., Relitti N., Brindisi M., Magnano S., Zisterer D., Gemma S., Butini S., Campiani G. (2020). Autophagy modulators for the treatment of oral and esophageal squamous cell carcinomas. Med. Res. Rev..

[13] Liu H., Xu X., Wu R., Bi L., Zhang C., Chen H., Yang Y. (2021). Antioral squamous cell carcinoma effects of carvacrol via inhibiting inflammation, proliferation, and migration related to Nrf2/Keap1 pathway. BioMed Res. Int..

[14] Lannagan T.R., Jackstadt R., Leedham S.J., Sansom O.J. (2021). Advances in colon cancer research: In vitro and animal models. Curr. Opin. Genet. Dev..

[15] Engstrom P.F., Arnoletti J.P., Benson A.B., Chen Y.J., Choti M.A., Cooper H.S., Covey A., Dilawari R.A., Early D.S., Enzinger P.C. (2009). Colon cancer. J. Natl. Compr. Cancer Netw..

[16] Fadel M.G., Malietzis G., Constantinides V., Pellino G., Tekkis P., Kontovounisios C. (2021). Clinicopathological factors and survival outcomes of signet-ring cell and mucinous carcinoma versus adenocarcinoma of the colon and rectum: A systematic review and meta-analysis. Discov. Oncol..

[17] Mohamed A.A.A., Hançerlioğullari A., Rahebi J., Rezaeizadeh R., Lopez-Guede J.M. (2024). Colon Cancer Disease Diagnosis Based on Convolutional Neural Network and Fishier Mantis Optimizer. Diagnostics.

[18] Das S., Biswas S., Paul A., Dey A. (2018). AI Doctor: An intelligent approach for medical diagnosis. Industry Interactive Innovations in Science, Engineering and Technology.

[19] Schmidhuber J. (2015). Deep learning in neural networks: An overview. Neural Netw..

[20] Ali M., Ali R. (2021). Multi-input dual-stream capsule network for improved lung and colon cancer classification. Diagnostics.

[21] Sakr A.S., Soliman N.F., Al-Gaashani M.S., Pławiak P., Ateya A.A., Hammad M. (2022). An efficient deep learning approach for colon cancer detection. Appl. Sci..

[22] Masud M., Sikder N., Nahid A.-A., Bairagi A.K., AlZain M.A. (2021). Machine learning approach to diagnosing lung and colon cancer using a dl-based classification framework. Sensors.

[23] Kumar N., Sharma M., Singh V.P., Madan C., Mehandia S. (2022). An empirical study of handcrafted and dense feature extraction techniques for lung and colon cancer classification from histopathological images. Biomed. Signal Process. Control.

[24] Mehmood S., Ghazal T.M., Khan M.A., Zubair M., Naseem M.T., Faiz T., Ahmad M. (2022). Malignancy detection in lung and colon histopathology images using transfer learning with class selective image processing. IEEE Access.

[25] Talukder A., Islam M., Uddin A., Akhter A., Hasan K.F., Moni M.A. (2022). Machine learning-based lung and colon cancer detection using deep feature extraction and ensemble learning. Expert Syst. Appl..

[26] Attallah O., Aslan M.F., Sabanci K. (2022). A Framework for Lung and Colon Cancer Diagnosis via Lightweight Deep Learning Models and Transformation Methods. Diagnostics.

[27] Akinyemi J.D., Akinola V., Adekunle V., Adetiloye V., Dansu E.J. (2023). Lung and colon cancer detection from CT images using Deep Learning. Mach. Graph. Vis..

[28] Borkowski A.A., Bui M.M., Thomas L.B., Wilson C.P., DeLand L.A., Mastorides S.M. (2019). Lung and colon cancer histopathological image dataset (LC25000). arXiv.

[29] Singh O., Singh K.K. (2023). An approach to classify lung and colon cancer of histopathology images using deep feature extraction and an ensemble method. Int. J. Inf. Tecnol..

[30] Bilal M., Maqsood M., Yasmin S., Ul Hasan N., Rho S. (2022). A transfer learning-based efficient spatiotemporal human action recognition framework for long and overlapping action classes. J. Supercomput..

[31] Zoph B., Vasudevan V., Shlens J., Le Q.V. Learning Transferable Architectures for Scalable Image Recognition. Proceedings of the 2018 IEEE/CVF Conference on Computer Vision and Pattern Recognition.

[32] Radhika K., Devika K., Aswathi T., Sreevidya P., Sowmya V., Soman K.P., Rout M., Rout J., Das H. (2020). Performance Analysis of NASNet on Unconstrained Ear Recognition. Nature Inspired Computing for Data Science.

[33] Tan M., Le Q. EfficientNet: Rethinking model scaling for convolutional neural networks. Proceedings of the 36th International Conference on Machine Learning.

[34] Alhichri H., Alswayed A.S., Bazi Y., Ammour N., Alajlan N.A. (2021). Classification of remote sensing images using efficientnet-b3 cnn model with attention. IEEE Access.

[35] Sandler M., Howard A., Zhu M., Zhmoginov A., Chen L.-C. MobileNetV2: Inverted residuals and linear bottlenecks. Proceedings of the 2018 IEEE/CVF Conference on Computer Vision and Pattern Recognition.

[36] Putra T.A., Rufaida S.I., Leu J. (2020). Enhanced skin condition prediction through machine learning using dynamic training and testing augmentation. IEEE Access.

[37] Mohammed A., Kora R. (2021). An effective ensemble deep learning framework for text classification. J. King Saud Univ.-Comput. Inf. Sci..

